# Asymptomatic and submicroscopic malaria infections in sugar cane and rice development areas of Ethiopia

**DOI:** 10.1186/s12936-023-04762-5

**Published:** 2023-11-08

**Authors:** Hallelujah Getachew, Assalif Demissew, Ashenafi Abossie, Kassahun Habtamu, Xiaoming Wang, Daibin Zhong, Guofa Zhou, Ming-Chieh Lee, Elizabeth Hemming-Schroeder, Lauren Bradley, Teshome Degefa, Dawit Hawaria, Arega Tsegaye, James W.Kazura, Cristian Koepfli, Guiyun Yan, Delenasaw Yewhalaw

**Affiliations:** 1 Department of Medical Laboratory Technology, Arbaminch College of Health Sciences, Arbaminch, Ethiopia; 2 Department of Medical Laboratory Sciences, College of Medicine and Health Sciences, Ambo University, Ambo, Ethiopia; 3 Department of Medical Laboratory Sciences, College of Medicine and Health Science, Arbaminch University, Arbaminch, Ethiopia; 4 Menelik II Medical & Health Science College, Addis Ababa, Ethiopia; 5 Program in Public Health, University of California at Irvine, Irvine, CA92697 USA; 6 Center for Vector Born Infectious Diseases (CVID), Department of Microbiology Immunology and Pathology, Colorado State University, Fort Collins, USA; 7 Department of Medical Laboratory Sciences, Institute of Health, Jimma University, Jimma, Ethiopia; 8 School of Environmental Health, Hawassa University, Hawassa, Ethiopia; 9 Department of Biology, College of Natural Science, Jimma University, Jimma, Ethiopia; 10 Biomedical Research Case Western Reserve University, Cleveland, OH USA; 11 Center for Global Health & Disease School of Medicine Case, Western Reserve University, Cleveland, OH USA; 12 Department of Biological Sciences 319 Galvin Life Sciences, Eck Institute for Global Health, University of Notre Dame, Notre Dame, USA; 13 Tropical and Infectious Diseases Research Center (TIDRC), Jimma University, Jimma, Ethiopia; 14 Aklilu Lemma Institute of Pathobiology, Addis Ababa University, Addis Ababa, Ethiopia; 15 Department of Microbial, Cellular & Molecular Biology, Addis Ababa University, Addis Ababa, Ethiopia

**Keywords:** Irrigation, Submicroscopic malaria, *P. ovale*, Ethiopia

## Abstract

**Background:**

Water resource development projects, such as dams and irrigation schemes, have a positive impact on food security and poverty reduction. However, such projects could increase prevalence of vector borne disease, such as malaria. This study investigate the impact of different agroecosystems and prevalence of malaria infection in Southwest Ethiopia.

**Methods:**

Two cross-sectional surveys were conducted in the dry and wet seasons in irrigated and non-irrigated clusters of Arjo sugarcane and Gambella rice development areas of Ethiopia in 2019. A total of 4464 and 2176 study participants from 1449 households in Arjo and 546 households in Gambella enrolled in the study and blood samples were collected, respectively. All blood samples were microscopically examined and a subset of microscopy negative blood samples (n = 2244) were analysed by qPCR. Mixed effect logistic regression and generalized estimating equation were used to determine microscopic and submicroscopic malaria infection and the associated risk factors, respectively.

**Results:**

Prevalence by microscopy was 2.0% (88/4464) in Arjo and 6.1% (133/2176) in Gambella. In Gambella, prevalence was significantly higher in irrigated clusters (10.4% vs 3.6%) than in non-irrigated clusters (*p* < 0.001), but no difference was found in Arjo (2.0% vs 2.0%; *p* = 0.993). On the other hand, of the 1713 and 531 samples analysed by qPCR from Arjo and Gambella the presence of submicroscopic infection was 1.2% and 12.8%, respectively. *Plasmodium falciparum, Plasmodium vivax,* and *Plasmodium ovale* were identified by qPCR in both sites. Irrigation was a risk factor for submicroscopic infection in both Arjo and Gambella. Irrigation, being a migrant worker, outdoor job, < 6 months length of stay in the area were risk factors for microscopic infection in Gambella. Moreover, school-age children and length of stay in the area for 1–3 years were significant predictors for submicroscopic malaria in Gambella. However, no ITN utilization was a predictor for both submicroscopic and microscopic infection in Arjo. Season was also a risk factor for microscopic infection in Arjo.

**Conclusion:**

The study highlighted the potential importance of different irrigation practices impacting on submicroscopic malaria transmission. Moreover, microscopic and submicroscopic infections coupled with population movement may contribute to residual malaria transmission and could hinder malaria control and elimination programmes in the country. Therefore, strengthening malaria surveillance and control by using highly sensitive diagnostic tools to detect low-density parasites, screening migrant workers upon arrival and departure, ensuring adequate coverage and proper utilization of vector control tools, and health education for at-risk groups residing or working in such development corridors is needed.

**Supplementary Information:**

The online version contains supplementary material available at 10.1186/s12936-023-04762-5.

## Background

Water resource development projects such as dams and irrigation schemes have a positive impact on food security and poverty reduction [1]. However, such projects also might alter the ecosystem and create favourable hatching ground for mosquito proliferation [2, 3]. They could result in a change in the malaria transmission pattern from seasonal to perennial, particularly in areas with seasonal and unstable malaria transmission [4, 5]. The job opportunity in irrigation projects attracts a large number of migrant workers who might be at increased risk of malaria. This is further exacerbated if migrant workers carry new strains such drug resistance parasites gene and also non-immune migrant works move to the area this may lead to malaria outbreak [6, 7].

Different agro-ecosystems and crop production types have an impact on mosquito proliferation, and consequently, the intensity of malaria transmission. Depending on the number of crop cycles, irrigation-based farming may also extend the mosquito breeding season and lengthen the malaria transmission period [8]. Major crop irrigation schemes in Africa include rice, sugarcane, cotton, wheat and vegetables. Among them, rice grown in flooded irrigation provides potential larval habitats [9]. Although a sugarcane plantation needs irrigation for maximum growth, it does not need to be flooded condition [10]. Furthermore, *Anopheles gambiae*, the major malaria vector in Africa, prefers environments with direct sunlight for breeding, thus the dense vegetation cover by sugarcane development would make the environment undesirable [8]. However, poorly maintained water canals used for sugarcane irrigation may produce hatching grounds for mosquitoes [11]. Studies have shown inconsistent results of malaria prevalence following the implementation of irrigation schemes. Several studies found that when compared to non-irrigated villages, the prevalence of malaria was increased in irrigated or dam areas [12–16]. Other studies have shown decreased malaria prevalence in irrigated areas [8, 17, 18]. Such discrepancies suggest that the effects of irrigation or dams on malaria transmission are poorly understood.

Irrigation activities [4, 5] coupled with population movement [19–21] might also affect the transmission dynamics of malaria. In such setting where seasonal and migrant workers are attracted, asymptomatic infections are important sources of malaria transmission and become a bottle neck for malaria control and elimination efforts [22, 23]. This is because these groups might carry gametocytes that contribute to the persistent transmission of malaria [24, 25]. Often, the majority of asymptomatic infections are submicroscopic and can only be detected using molecular methods [26]. In endemic areas, malaria elimination may not be feasible with the existing diagnostic and intervention tool alone [25, 27]. Thus, additional strategies to detect asymptomatic carriers need to be considered. Therefore, this study assessed the relationships between two different agroecosystems and prevalence of malaria infection in sugarcane growing (Arjo) and rice growing (Gambella) areas of Southwest, Ethiopia.

## Methods

### Study area

This study was carried out in two areas of Southwest Ethiopia: Arjo (sugarcane growing) and Gambella (rice growing) areas. Arjo Didessa Sugarcane development irrigation is located in two districts of Buno Bedele Zone, namely Bedele and Dabo Hanna and one district of East Wellega, Jimma Arjo district. It is 540 km Southwest of Addis Ababa and is located at latitude 8.6°N, longitude 36.4^o^E, and an altitude with a ranges of 1300 to 2280 m. The mean annual rainfall is 1477 mm, with bimodal rainfall (long rainy season June to September and short rainy season February to March) and with a relatively short dry season between December to January. The estimated total population of Jimma Arjo, Dabo Hanna and Bedele districts was 271,378 [28].

**Fig. 1:**
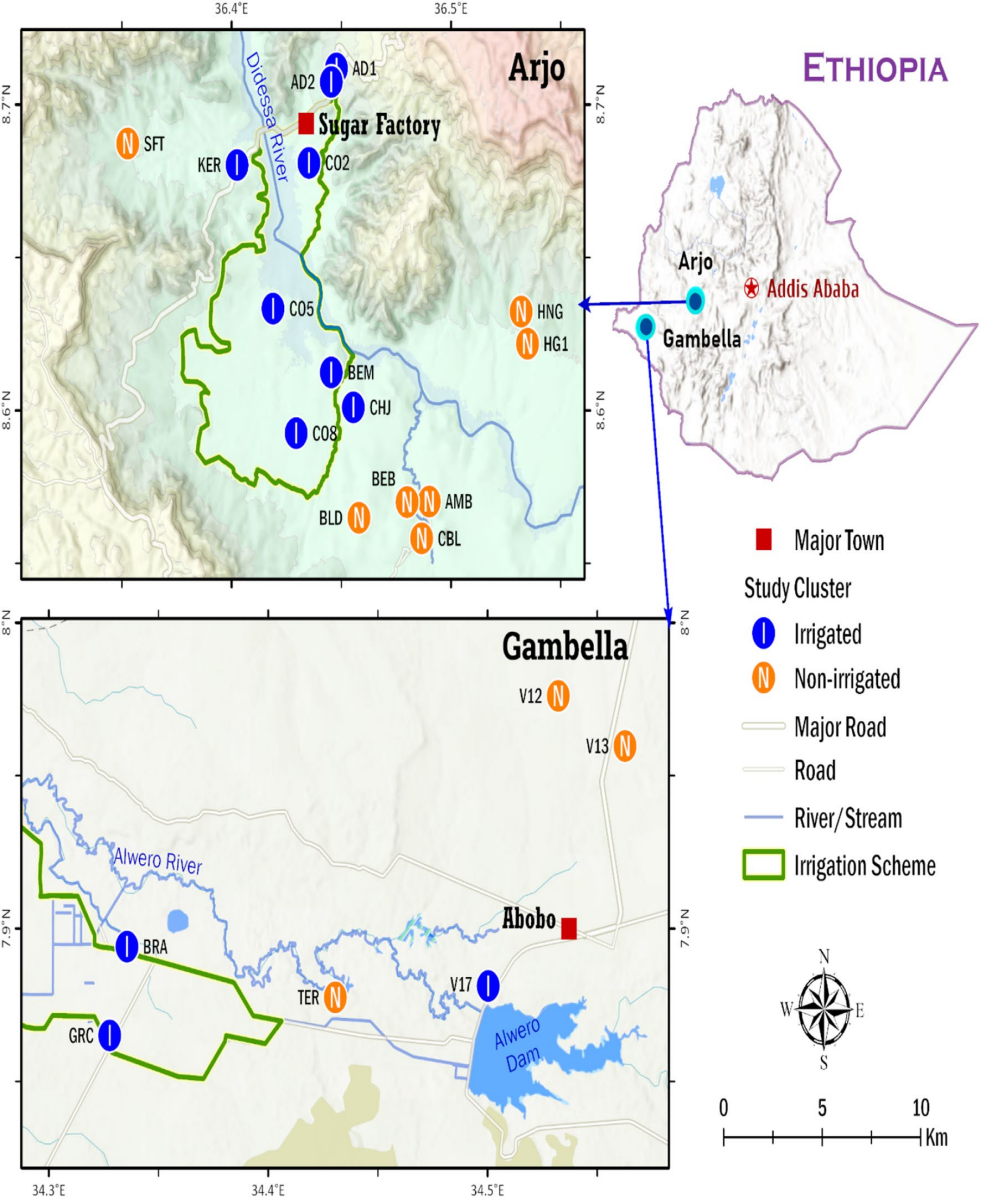
Map of the study sites and respective *kebele*

In Arjo, the sugarcane plantation irrigated by the use of sprinklers and in some part open gravity fed earthen ditches from Didessa River. While, the non-irrigation clusters, rain-fed farming took place, i.e. maize, sorghum, nut and peppers were grown at a subsistence level; and the villagers also keep cattle for their livelihood. Seven *kebeles* from irrigated and six *kebeles* from non-irrigated areas were selected as shown in Fig. 1 (upper section) and Additional file 1: Table S1.

The rice irrigation (owned by Saudi Star Agricultural Development PLC) is located in Abobo district of Gambella region in West Ethiopia. It is 811 km west of Addis Ababa located at latitude 7.9°N and longitude 34.5^o^E, and an altitude with a range of 400–500 m. The annual rainfall in Abobo is ranges between 900 to 1200 mm, with unimodal rainfall (long rainy season May to October) and long dry and hot season occurs between November–April [29].

The total population of Abobo district was 26,080 [28] living mainly on subsistence farming and fishing. Open gravity-fed earthen ditches from the main canal of Alwero dam supply the rice irrigation. The non-irrigated *kebeles* used rain-fed farming, cotton, maize, sorghum and fishing for their livelihood. Three rice irrigation *kebeles* and three non-irrigated *kebeles* were selected (Fig. 1, lower section) and Additional file 1: Table S1.

### Sample size and sampling

The sample size (n) calculation was done using the formula for estimating single proportion. In sugarcane growing (Arjo), the sample size was calculated at 95% confidence interval (CI), Z α/2 = 1.96, 1% marginal error (d), design effect (D_eff_) = 2, malaria prevalence of 2.6% [30] and 10% non-response rate. While, in rice growing areas (Gambella) the sample size was calculated at 95% CI, Z α/2 = 1.96, 2% marginal error (d), D_eff_ = 2 and malaria prevalence of 6.0% [31] with 10% non-response rate. Therefore, 2236 and 1083 study participants, respectively from sugarcane growing areas (Arjo) and rice growing areas (Gambella) were estimated in each cross-sectional surveys.

A cluster was defined as a village within a *kebele* comprising 100–200 households. A total 13 *kebeles* (small administrative unit) from Arjo (sugarcane growing) and 6 *kebeles* from Gambella (rice growing) were selected for this study. In Arjo (sugarcane growing), only one cluster was selected in each 11 *kebeles*. However, due to large population size two clusters were selected from Abote Didessa and Hunde Gudina *kebeles* (Fig. 1). Overall, 15 clusters were selected from Arjo (sugarcane growing). In Gamblella (rice growing), six clusters were selected from six *kebeles*. Study households for cross-sectional parasitological survey (CPS) were identified through systematic sampling of every fifth house. All consenting individuals living in the selected households were included in the study.

### Demographic and parasitological survey

These demographic and parasitological surveys are a part of wider study on environmental modification in relation to malaria and conducted in parallel with entomological surveillance. The entomological studies result are reported elsewhere [3, 32]. Demographic data and capillary blood samples were collected simultaneously in eight irrigated and seven non-irrigated clusters of sugarcane growing (Arjo) and in three irrigated and three non-irrigated clusters of rice growing (Gambella) (Fig. 1). Irrigated clusters were within 1 km (km) radius and non-irrigated clusters were above 1 km radius from the edge of irrigated areas, considering *Anopheles* mosquito flight range [33]. The spatial coordinates and data were collected using Open Data Kit (ODK) (https://opendatakit.org/about/research/). Surveys were conducted in March 2019 (dry season) and October 2019 (wet season), representing low and high malaria transmission season, respectively. Socio-demographic characteristics, mosquito preventive measures and village Global Positioning System (GPS) coordinate were collected in both surveys. As well, malaria symptoms such as fever and any malaria related symptoms (chills, sweating, headache, vomit, and abdominal pain) during or 48 h prior to blood collection. Study participant with body temperature above 37 °C during or 48 h prior to blood collection considered as febrile.

Capillary blood samples (300 μL) were collected from all consenting participants for diagnosis by CareStart^™^ Malaria Pf/Pv (HRP2/pLDH) Ag Combo RDT test kits (Access Bio Ethiopia, INC.) and microscopy. Quantitative polymerase chain reaction (qPCR) was performed for randomly selected smear negative blood samples. For qPCR, dried blood spot (DBS) were prepared. The dried blood smear slides and the four dots of DBS samples were packed in slide box and plastic bag with desiccant, respectively. All samples were stored at room temperature then transported to Jimma University diagnostic and parasite culture laboratory for microscopic examination, DNA extraction and qPCR parasite determination.

### Outcome and predictor variables

The primary outcome of the study was microscopic and submicroscopic parasite prevalence. While, individual and cluster level factors used to explain outcomes. Individual level factors were age, sex, occupation, migrant worker, ITN utilization, and duration of stay in the area. Two sets of cluster level factors were considered in the analysis; location factors (irrigated or non-irrigated area, survey season) and household factors (house constructed material, number of sleeping rooms, indoor residual spraying (IRS), and insecticide-treated net (ITN) ownership with the household).

In this study, occupation was classified into two categories: indoor and outdoor jobs. The indoor category consisted of office workers, housewives, non-schooled children, and students. The outdoor category comprised agricultural workers, herdsman, factory workers (sugarcane and rice plantation workers), miners, fisherman, drivers, construction workers, and day or night watchmen. On the other hand, migrant workers are workers who stayed in the area for 1 to 6 months. They were two types in the study area: seasonal and casual workers. Seasonal workers come to the area during the planting and harvesting season and work for up to 3 months under a contract. Casual workers work on a weekly basis.

### Microscopic examination

Thick and thin blood films were prepared and stained with 10% Giemsa solution and a BSc medical laboratory technologist examined under light microscope at Jimma University. Blood films were considered negative if no parasites were detected in 200 fields of the thick blood film. Thick blood films were used for detection and quantification of the parasites. Thin blood films were used for species identification. Gametocytes and asexual stage parasites were counted against 200 and 500 white blood cells (WBCs), respectively, and densities (parasite/µl) was estimated using a factor of 8000 leukocytes/µl [34, 35]. A second medical laboratory technician rechecked all positive slides and 10% of all negative slides.

### DNA extraction

Modified Chelex-100 resin was used for DNA extraction protocol [36]. One dot of the DBS was cut into piece of approximately 3–5 mm using a puncher. The puncher was cleaned by punching paper ten times after every sample. The blotted filter paper was transferred to 1.5 ml Eppendorf tube then 950 µl phosphate buffered saline (PBS) and 50 µl 10% Saponin were added for the lysis of RBCs. After mixing the sample, it was incubated at 4 °C for 4 h or overnight. The mixture was centrifuged at 14,000 rpm for 10 min at room temperature and the supernatant was discarded. Any remnant of Saponin was removed by adding 1 ml of PBS and centrifuge at 14,000 rpm for about 5 min. The remaining PBS was discarded by centrifugation of the tube for about 15 s. The filter paper was left for 15 min to dry at room temperature. Subsequently, 150 µl 20%Chelex suspension and 100 µl distilled water was added to extract the parasite DNA by incubating the mixture at 95 °C in a water bath for 10 min with vortexing the mixture every 2 min during the process of incubation. Finally, the mixture was centrifuged at 14,000 rpm for 1 min and 200 µl supernatant (DNA) was transferred to a 0.5 ml tube and stored at − 20 °C for qPCR analysis.

### *Plasmodium* species identification by multiplex qPCR

Genomic DNA of each sample was amplified using 18S rRNA genes based primers. Amplification was performed in a 12 μl qPCR reaction mixture containing 2 μl genomic DNA, 6 μl PerfeCTa (2X), 0.4 μl forward and reverse primers for *Plasmodium falciparum*, *Plasmodium vivax* and *Plasmodium ovale*, each (F-F, F-R, Pv-1, Pv-2, Po-1, and Po-2) then 0.5 μl Pf-fam, 0.5 μl Pv-vic, and 0.5 μl Po-Ned for with *P. falciparum*, *P. vivax* and *P. ovale* TaqMan probes in 0.1 double distilled water [37, 38] since *P. ovale* has been reported in Northwest and Southeastern, Ethiopia. Amplification reactions was performed in a 96 well Quant Studio 3, Applied Biosystems Real-Time PCR, with a hold stage at 50 °C for 2 min and 95 °C for 2 min, followed by 45 cycles of qPCR amplification stage at 95 °C for 3 s and 60 °C for 30 s.

### Data processing and analysis

Data was collected using ODK and uploaded to a server then downloaded in a comma-separated values (CSV) file. The CSV file converted in to Excel for data cleaning and exported to STATA (version 15) and R (version 4.3.1) for data analysis. Variance Inflation Factor (VIF) test was used to check multicollinearity between independent variables. Multilevel logistic regression models were fitted to identify individual and cluster-level risk factors of *Plasmodium* infection by microscopy in Arjo and Gambella areas, Southwest Ethiopia.

In this study, both univariate and multivariate multilevel modelling was performed using mixed-effects logistic regression for microscopic *Plasmodium* infection. In univariate multilevel, the relationship of each variable through entering household, higher group, was analysed. This model gives accurate standard errors to deal with biases that linear regression and descriptive analysis, which are used to find risk factors, do not account for. To overcome this limitation, four models were developed to find risk factors of microscopic *Plasmodium* infection in this study area.

A null model with no explanatory variables (by incorporating only household specific random effect) was fitted to measure the outcome variable’s variance and to show the probability of using subsequent models. Model I and II were adjusted to individual and cluster level risk factors respectively. The full model was set to include both individual and cluster-level factors. Only variables with 95% CI significance and *p*-value below 0.05 from the full model were retained for discussion. The adjusted odds ratio (AOR) was used to show the association between microscopic *Plasmodium* infection and predictor variables at the individual and cluster-level. Variance at matching 95% CI for each model was used to measure variation due to the effects of risk factors at different levels. Variance partition coefficient (VPC) or inter-class correlation (ICC) was used to find the percentage variance accounted for by the risk factors at different levels. In this study VPC was calculated based on the latent variable method, which is $\mathrm{VPC}=\frac{{\widehat{\uptau }}^{2}}{{\widehat{\uptau }}^{2}+{\frac{\uppi }{3}}^{2}}$ where,${\widehat{\tau }}^{2}$ is the estimated variance of the distribution of the random effects [39] and ${\frac{\pi }{3}}^{2}$=3.29 (the variance of a logistic distribution with scale) [40].

The median odds ratio (MOR) was used to measure unaccounted variation between each model that is always greater or equal to one and no variation when MOR is one. The Akaike information criterion (AIC) was also used to compare how well each model fit to the data, with a lower value indicates best fit. However, logistic generalized estimating equation (GEE) was used for submicroscopic *Plasmodium* infection. GEE is among the generalized linear model used to analysed repeated measurements [41]. Repeated data are usually correlated to each other. GEE help in adjusting for these correlations that occur within a subject. In this study, submicroscopic malaria was dichotomized that follows a binomial distribution. The specified probability distribution was binomial with logit link function and the working correlation matrix structure was exchangeable. The covariance matrix was robust estimator, and the scale parameter was Person chi-square. Univariate and multivariate analysis were done to measure the risk factors of submicroscopic malaria infection, and result was reported as odds ratio. Median comparisons for parasite density between symptomatic and asymptomatic individuals were made using Mann–Whitney test.

## Results

Demographic information was obtained from 6640 individuals including 4464 (67.2%) from 1449 households in Arjo and 2176 (32.8%) from 546 households in Gambella and can be found in Tables 1, 2. In sugarcane growing (Arjo), the median age of the study participants were 20 years with a minimum 6 months and maximum 99 years. More than half 53.6% (2394/4464) of the study participants were female. Half 50.0% (2230/4464) of the study participants were from irrigated clusters. While, in rice growing (Gambella) the median age of the study participants were 21 years with a minimum 6 months and maximum 93 years. More than half 54.1% (1178/2176) of the study participant were male. Of the total study participants only 800 (36.8%) belonged to irrigated clusters.

**Table 1:** Prevalence of *Plasmodium* infection and risk factors in Arjo (March 2019 and October2019)

Factors	Dry season					Wet season			
		Microscopy		PCR		Microscopy		PCR	
		Total	Positive n (%)	Total	Positive n (%)	Total	Positive n (%)	Total	Positive n (%)
***Individual level factors***		**1839**	**6 (0.3)**	**529**	**9 (1.7)**	**2625**	**82 (3.1)**	**1184**	**12 (1.0)**
Sex	Female	982	4 (0.4)	281	3 (1.1)	1412	43 (3.0)	638	6 (0.9)
	Male	857	2 (0.2)	248	6 (2.4)	1213	39 (3.2)	546	6 (1.1)
Age group (in years)	< 5	221	1 (0.5)	53	1 (1.9)	414	13 (3.1)	191	3 (1.6)
	5_15	422	0 (0.0)	108	0 (0.0)	673	20 (3.0)	282	2 (0.7)
	> 15	1196	5 (0.4)	368	8 (2.2)	1538	49 (3.2)	711	7 (1.0)
Job	Indoor	854	3 (0.4)	230	2 (0.9)	1393	40 (2.9)	612	7 (1.1)
	Outdoor	985	3 (0.3)	299	7 (2.3)	1232	42 (3.4)	572	5 (0.9)
Length of stay in the area	< 6 months	142	1 (0.7)	49	0 (0.0)	93	4 (4.3)	41	0 (0.0)
	7–12 months	69	1 (1.4)	22	0 (0.0)	140	7 (5.0)	60	0 (0.0)
	1–3 years	191	0 (0.0)	55	2 (3.6)	262	8 (3.1)	131	1 (0.8)
	> 3 years	1437	4 (0.3)	403	7 (1.7)	2130	63 (3.0)	952	11 (1.2)
Migrant worker	No	1679	5 (0.3)	476	9 (1.9)	2528	79 (3.1)	1138	12 (1.1)
	Yes	160	1 (0.6)	53	0 (0.0)	97	3 (3.1)	46	0 (0.0)
ITN utilization	Every night	150	0 (0.0)	50	1 (2.0)	1865	37 (2.0)	876	2 (0.2)
	Sometimes	658	1 (0.2)	171	3 (1.8)	111	3 (2.7)	46	1 (2.2)
	No use	1031	5 (0.5)	308	5 (1.6)	649	42 (6.5)	262	9 (3.4)
***Cluster level factors***									
Irrigation status	Irrigated	977	2 (0.2)	342	6 (1.8)	1253	42 (3.4)	581	12 (2.1)
	Non-irrigated	862	4 (0.5)	187	3 (1.6)	1372	40 (2.9)	603	0 (0.0)
HH education level	≥ Secondary	256	1 (0.4)	80	2 (2.5)	335	8 (2.4)	177	1 (0.6)
	Primary	452	1 (0.2)	142	1 (0.7)	721	14 (1.9)	329	6 (1.8)
	No education	1131	4 (0.4)	307	6 (2.0)	1569	60 (3.8)	678	5 (0.7)
Family size	< 5	1061	5 (0.5)	315	6 (1.9)	1317	36 (2.7)	604	5 (0.8)
	> 5	778	1 (0.1)	214	3 (1.4)	1308	46 (3.5)	580	7 (1.2)
*House construction material*									
Roof material	Corrugated iron	1200	4 (0.3)	332	6 (1.8)	1732	51 (2.9)	761	7 (0.9)
	Thatch	639	2 (0.3)	197	3 (1.5)	893	31 (3.5)	423	5 (1.2)
Wall material	Corrugated iron	298	2 (0.7)	103	2 (1.9)	277	8 (2.9)	133	1 (0.8)
	Mud/cement	1541	4 (0.3)	426	7 (1.6)	2348	74 (3.2)	1051	11 (1.0)
No. of sleeping room	≥ Three	224	1 (0.4)	60	0 (0.0)	476	14 (2.9)	205	1 (0.5)
	Two	939	2 (0.2)	260	6 (2.3)	1219	41 (3.4)	520	5 (1.0)
	One	676	3 (0.4)	209	3 (1.4)	930	27 (2.9)	459	6 (1.3)
*Vector control measures*									
No. of ITN/house	≥ Three	252	0 (0.0)	70	0 (0.0)	673	22 (3.3)	293	0 (0.0)
	Two	503	0 (0.0)	112	0 (0.0)	930	25 (2.7)	401	2 (0.5)
	One	407	1 (0.2)	129	4 (3.1)	674	14 (2.1)	336	1 (0.3)
	Not available	677	5 (0.7)	218	5 (2.3)	348	21 (6.0)	154	9 (5.8)
IRS sprayed	No	1167	2 (0.2)	322	6 (1.9)	1533	52 (3.4)	691	4 (0.6)
	Yes	672	4 (0.6)	207	3 (1.4)	1092	30 (2.7)	493	8 (1.6)
*Household*		843	6 (0.7)	358	8 (2.2)	958	72 (7.5)	702	11 (1.6)

**Table 2:** Prevalence of *Plasmodium* infection and individual risk factors in Gambella (March 2019 and October2019)

Factor	Dry season					Wet season			
		Microscopy		PCR		Microscopy		PCR	
		Total	Positive n (%)	Total	Positive n (%)	Total	Positive n (%)	Total	Positive n (%)
***Individual level factors***		**1337**	**86 (6.4)**	**294**	**25 (8.5)**	**839**	**47 (5.6)**	**237**	**43 (18.1)**
Sex	Female	605	34 (5.6)	137	12 (8.8)	393	18 (4.6)	94	17 (18.1)
	Male	732	52 (7.1)	157	13 (8.3)	446	29 (6.5)	143	26 (18.2)
Age group (in years)	< 5	132	2 (1.5)	14	3 (21.4)	111	5 (4.5)	24	2 (8.3)
	5_15	361	6 (1.7)	65	9 (13.8)	217	10 (4.6)	41	11 (26.8)
	> 15	844	78 (9.2)	215	13 (6.0)	511	32 (6.3)	172	30 (17.4)
Job	Indoor	695	15 (2.2)	110	14 (12.7)	449	19 (4.2)	99	15 (15.2)
	Outdoor	642	71 (11.1)	184	11 (6.0)	390	28 (7.2)	138	28 (20.3)
Length of stay in the area	< 6 months	23	5 (21.7)	3	2 (66.7)	25	3 (12.0)	6	0 (0.0)
	7–12 months	33	4 (12.1)	2	0 (0.0)	33	2 (6.1)	8	3 (37.5)
	1–3 years	60	5 (8.3)	13	5 (38.5)	27	1 (3.7)	9	3 (33.3)
	> 3 years	1221	72 (5.9)	276	18 (6.5)	754	41 (5.4)	214	37 (17.3)
Migrant worker	No	1175	46 (3.9)	217	20 (9.2)	752	39 (5.2)	172	32 (18.6)
	Yes	162	40 (24.7)	77	5 (6.5)	87	8 (9.2)	65	11 (16.9)
ITN utilization	Every night	1007	55 (5.5)	199	14 (7.0)	476	18 (3.8)	138	28 (20.3)
	Sometimes	46	2 (4.3)	12	0 (0.0)	11	1 (9.1)	1	1 (100.0)
	No use	284	29 (10.2)	83	11 (13.3)	352	28 (8.0)	98	14 (14.3)
***Cluster level factors***									
Irrigation status	Irrigated	472	61 (12.9)	123	15 (12.2)	328	22 (6.7)	89	22 (24.7)
	Non-irrigated	865	25 (2.9)	171	10 (5.8)	511	25 (4.9)	148	21 (14.2)
HH education level	≥ Secondary	474	34 (7.2)	111	11 (9.9)	231	10 (4.3)	69	18 (26.1)
	Primary	360	17 (4.7)	87	6 (6.9)	235	14 (6.0)	70	10 (14.3)
	No education	503	35 (7.0)	96	8 (8.3)	373	23 (6.2)	98	15 (15.3)
Family size	< 5	783	65 (8.3)	192	14 (7.3)	434	23 (5.3)	144	26 18.1)
	> 5	554	21 (3.8)	102	11 (10.8)	405	24 (5.9)	93	17 (18.3)
*House construction material*									
Roof material	Corrugated iron	664	58 (8.7)	159	13 (8.2)	488	25 (5.1)	141	30 (21.3)
	Thatch	673	28(4.2)	135	12 (8.9)	351	22 (6.3)	96	13 (13.5)
Wall material	Corrugated iron	176	37 (21.0)	81	6 (7.4)	132	7 (5.3)	86	17 (19.8)
	Mud/cement	1161	49 (4.2)	213	19 (8.9)	707	40 (5.7)	151	26 (17.2)
No. of sleeping room	≥ Three	87	2 (2.3)	19	3 (15.8)	134	5 (3.7)	23	7 (30.4)
	Two	254	9 (3.5)	37	4 (10.8)	271	15 (5.5)	53	12 (22.6)
	One	996	75 (7.5)	238	18 (7.6)	434	27 (6.2)	161	24 (14.9)
*Vector control measures*									
No. of ITN/house	≥ Three	232	10 (4.3)	45	3 (6.7)	41	2 (4.9)	10	4 (40.0)
	Two	443	13 (2.9)	76	4 (5.3)	207	5 (2.4)	47	10 (21.3)
	One	422	40 (9.5)	97	8 (8.2)	264	13 (4.9)	89	15 (16.9)
	Not available	240	23 (9.6)	76	10 (13.2)	327	27 (8.3)	91	14 (15.4)
IRS sprayed	No	713	49 (6.9)	199	14 (7.0)	320	20 (6.3)	125	24 (19.2)
	Yes	624	37 (5.9)	95	11 (11.6)	519	27 (5.2)	112	19 (17.0)
*Household*		388	70 (18.0)	210	22 (10.5)	297	40 (13.5)	174	41 (23.6)

### Parasite prevalence in sugarcane growing (Arjo) area

**Fig. 2:**
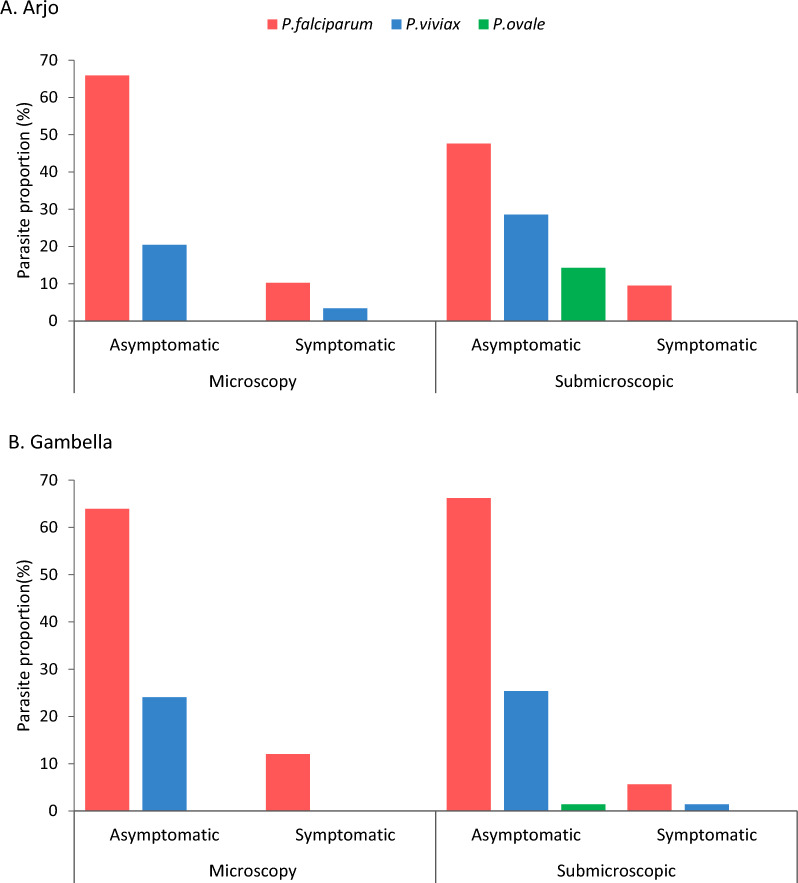
Proportion of *Plasmodium* infections among asymptomatic and symptomatic by parasite species in sugarcane growing Arjo **A** and rice-growing Gambella **B**, Ethiopia. (Individuals with malaria related symptoms or asymptomatic during or 48 h prior to mass blood survey)

Of 4464 blood samples collected in Arjo, the overall malaria prevalence by microscopy was 2.0% (88/4464). The proportion of *Plasmodium* species by microscopy was 76.1% (67/88) *P. falciparum* and 23.9% (21/88) *P. vivax*. The majority, 86.4% (76/88), of the *Plasmodium* positive individuals were asymptomatic (Fig. 2). Malaria prevalence by microscopy showed no significant difference between sugarcane irrigated 2.0% (44/2230) and in non-irrigated clusters 2.0% (44/2234) (*p* = 0.993). However, the prevalence of malaria differs by season, in the wet season higher malaria prevalence was observed than in the dry season in both the sugarcane irrigated (wet: 3.3% (42/1253); dry: 0.2% (2/977); *p* = 0.0001) and non-irrigated clusters (wet: 2.9% (40/1372); dry: 0.5% (4/862); *p* = 0.0001) (Table 1).

**Fig. 3:**
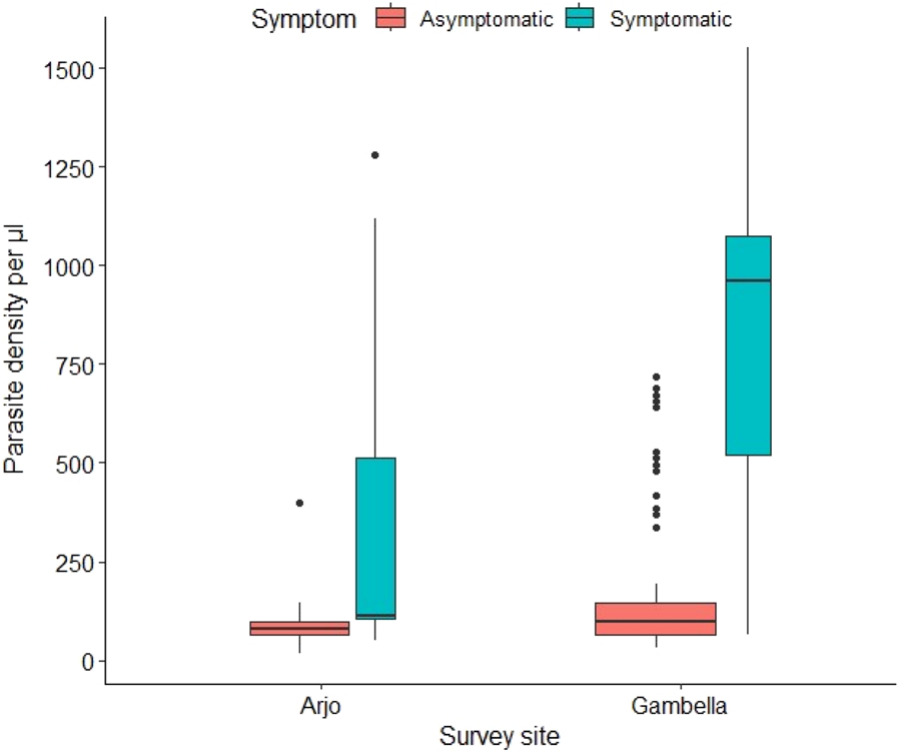
Parasite density in symptomatic and asymptomatic *Plasmodium-*infections by microscopy in Arjo and Gambella, Ethiopia

There was significant difference in parasitemia load between symptomatic and asymptomatic *Plasmodium* infection by microscopy. The median parasite density/μl for symptomatic and asymptomatic infection in sugarcane growing (Arjo) was 112, IQR: 88–672 and 80, IQR: 64–96, respectively with *p* = 0.0015) (Fig. 3). This indicates there was an evidence of a difference in parasite density between symptomatic and asymptomatic microscopically detected malaria.

One thousand seven hundred thirteen (1713) samples were analysed by qPCR. Of the total samples analysed by qPCR, 1.2% (21/1713) carried submicroscopic infections. The majority 90.5% (19/21) were asymptomatic and 9.5% (2/21) were symptomatic during or 48 h prior to the survey. The number of *Plasmodium* species in submicroscopic malaria infection was 12 *P. falciparum*, 6 *P. vivax*, and 3 *P. ovale* (Fig. 2). The identified rare species (*P. ovale*) was from irrigated cluster. The prevalence of submicroscopic malaria was high in irrigated cluster than non-irrigated (Table 1).

### Parasite prevalence in rice growing (Gambella) area

The overall malaria prevalence by microscopy was 6.1% (133/2176) in rice growing areas (Gambella). The proportion of *Plasmodium* species by microscopy was 75.9% (101/133) *P. falciparum* and 24.1% (32/133) *P. vivax*. Out of total positive by microscopy, 117(88.0%) were asymptomatic (Fig. 2).

The prevalence of microscopically detected asymptomatic malaria was 10.4% (83/800) and 3.6% (50/1376) in rice irrigated and non-irrigated clusters, respectively (*p* = 0.0001, Table 2). In rice irrigated clusters prevalence was higher during the dry season than in the wet season [12.9% (61/472) vs 6.7% (22/328); *p* = 0.0046]. In non-irrigated clusters, marginally significant difference in malaria prevalence was observed between the wet season [4.9% (25/511)] and dry season [2.9% (25/865), *p* = 0.055] (Table 2). In addition, increase prevalence observed by migrant workers status, age group, job type, the length of stay in the area and ITN utilization during dry season (Table 2).

There was significant difference in parasitemia load between symptomatic and asymptomatic *Plasmodium* infection by microscopy. The median parasite density/μl for symptomatic and asymptomatic infection in Gambella was 960, IQR: 280–1084 and 96, IQR: 64–144, respectively with *p* < 0.0001) (Fig. 3). This also indicates there was an evidence of a difference in parasite density according to the Mann–Whitney test (*p* < 0.0001).

Of the 531 analysed by qPCR, 12.8% (68/531) were submicroscopic malaria infection. Nine-tenth 92.6% (63/68) of the positive individuals were asymptomatic and 7.4% (5/68) were symptomatic. Among submicroscopic infections, 48 were *P. falciparum*, 16 *P. vivax*, three mixed (*P. falciparum* and *P. vivax*), and one *P. ovale* (Fig. 2). The identified *P. ovale* was from rice-irrigated cluster of Gambella. Irrigation, season, age and length of stay in the area showed higher proportion of submicroscopic malaria infection (Table 2).

### Multivariate analysis of risk factors for *Plasmodium* infection

In sugarcane growing (Arjo), the multilevel logistic regression of the fixed effect analysis indicated, survey season and ITN utilization were associated with *Plasmodium* infection by microscopy. Survey seasons was strong predictor of microscopically detected malaria infection [AOR: 21.06, 95%CI 7.94–55.84, *p* = 0.0001]. Similarly individuals who did not use ITN had higher microscopically detected malaria infection than those who use ITN daily [AOR: 5.09, 95%CI 2.33–11.12, *p* = 0.0001] (Table 3). In addition, logistic GEE analysis of submicroscopic infection showed individual from irrigated cluster were 4.9 times higher risk of submicroscopic infection than from the non- irrigated clusters [AOR: 4.89, 95%CI 1.33–17.91, *p* = 0.017], study participants who did not use ITN were 7.36 times higher risk of submicroscopic infection than their counterparts of who used ITN regularly [AOR: 7.36, 95%CI 2.02–26.72, *p* = 0.002] and sometimes ITN utilization were also significant predictors of submicroscopic malaria [AOR:6.56, 95%CI 1.29–33.24, *p* = 0.023] (Table 4). The univariate analysis attached in Additional file materials (S2, S3 and S4).

**Table 3:** Predictors of malaria risk obtained from multivariate mixed-effects logistic regression analysis by microscopy in Arjo area, Southwest Ethiopia: March 2019 and October 2019

	Sugarcane growing (Arjo)		
	Model-I	Model-II	Full model
Fixed effect	AOR,95%CI	AOR,95%CI	AOR,95%CI
Sex			
Male	1.00 [0.63,1.59]		1.05 [0.65,1.68]
Female	Ref.		Ref.
Age groups(in years)			
< 5	1.03 [0.52,2.07]		0.81 [0.39,1.68]
5–15	0.93 [0.51,1.70]		0.77 [0.41,1.44]
> 15	Ref		Ref
Duration of stay in the area			
3 years	Ref.		Ref.
1–3 years	0.84 [0.35,2.01]		0.98 [0.39,2.48]
7–12 months	2.11 [0.81,5.45]		2.17 [0.80,5.87]
< 6 months	0.93 [0.33,2.61]		1.81 [0.58,5.67]
ITN utilization			
Every night	Ref.		Ref.
Sometimes	0.24 [0.08,0.75]**		1.42 [0.39,5.11]
Never	1.67 [0.99,2.81]		5.09 [2.33,11.12]***
Irrigation status			
Non-irrigated		Ref	Ref
Irrigated		0.92 [0.53,1.59]	1.04 [0.59,1.83]
Season			
Dry		Ref.	Ref.
Wet		17.24 [6.60,45.00]***	21.06 [7.94,55.84]***
Family size			
< 5		Ref.	Ref.
> 5		1.26 [0.70,2.26]	1.32 [0.73,2.41]
Number of ITN per household			
≥ Three		Ref.	Ref.
Two		0.82 [0.38,1.77]	0.69 0.31,1.51]
One		0.69 [0.29,1.65]	0.59 [0.24,1.42]
Not available		2.90 [1.28,6.60]**	0.70 [0.26,1.90]

*p* <  = 0.01, **: *p* < 0.001***

**Table 4:** GEE model for submicroscopic malaria infection and risk factors in Arjo and Gambella, Ethiopia: March 2019 and October 2019

	Arjo (Sugarcane growing)	Gambella (Rice-growing)
Characteristics	AOR [95%CI]	AOR [95%CI]
Sex		
Male	1.48 [0.57–3.82]	0.86 0.49–1.49]
Female	Ref.	Ref.
Age groups(in year)		
< 5	1.29 [0.41–4.10]	0.44 [0.13–1.53]
5–15	0.41 [0.09–1.80]	3.72 [1.88–7.34]***
> 15	Ref.	Ref.
ITN utilization		
Every night	Ref.	
Sometimes	6.56 [1.29–33.24]*	
Never	7.36 [2.02–26.72]***	
Irrigation status		
Non-irrigated	Ref.	Ref.
Irrigated	4.89 [1.33–17.91]**	3.11 [1.56–6.17]***
Duration of stay in the area		
> 3 years		Ref.
1–3 years		10.19 [3.65–28.47]***
7–12 months		5.34 [0.69–41.19]
< 6 months		3.58 [0.54–23.59]
Season		
Dry		0.36 [0.20–0.65]***
Wet		Ref.
Household head level of education		
≥Secondary		Ref.
Primary		0.58 [0.28–1.19]
No education		0.71 [0.34–1.45]
Number of sleeping rooms		
≥Three		Ref.
Two		0.56 [0.23–1.32]
One		0.32 [0.14–0.71]**

*p* < 0.05*, *p* < 0.01**, *p* < 0.001***

In rice growing areas (Gambella) being a migrant worker, length of stay in the area, irrigated cluster, type of occupation and household construction material were significantly associated with *Plasmodium* infection by microscopy analysis. Being a migrant worker increased microscopically detected malaria infection by 14.7 times [AOR: 14.68, 95%CI 2.61–82.67, *p* = 0.002]. Microscopically detected malaria risk also increased with the length of stay in the area, individual who stayed < 6 months was 7 times higher risk than those stayed longer than 3 years [ AOR: 6.99, 95%CI: 1.68–29.00, *p* = 0.007]. Similarly, individuals who stayed seven to 12 months more likely to be infected with *Plasmodium* than those who stayed longer than 3 years [AOR: 5.13, 95%CI 1.26–20.92, *p* = 0.023]. In addition, irrigation [AOR: 4.09, 95%CI 1.82–9.16, *p* = 0.001], individual involved in outdoor jobs were 3 times risk for microscopically detected malaria infection [AOR: 2.93, 95%CI 1.36–6.32, *p* = 0.006], however, homes constructed with corrugated iron walls [AOR: 0.10, 95%CI 0.02–0.52, *p* = 0.006] were significant malaria predictors (Table 5). According to the logistic GEE analysis rice irrigation increased three-fold the risk of submicroscopic infection than non-irrigated [AOR: 3.11, 95%CI 1.56–6.18, *p* = 0.001]. Compared to adult older than 15 years of age, school-age children had a 3.7-fold higher submicroscopic infection [AOR: 3.72, 95% CI 1.88–7.34, *p* = 0.0001] while, one to 3 years length of stay in the area were [AOR: 10.19, 95%CI 3.65–28.47, *p* = 0.0001] risk factor for submicroscopic infection (Table 4). On the other hand, survey season and number of room were 64% and 68% less likely to have submicroscopic infection, respectively.

**Table 5:** Predictors of malaria risk obtained from multivariate mixed-effects logistic regression analysis by microscopy in Gambella area, Southwest Ethiopia: March 2019 and October 2019

	Rice-growing (Gambella)		
	Model-I	Model-II	Full model
Fixed effect	AOR,95%CI	AOR,95%CI	AOR,95%CI
Sex			
Male	0.81 [0.51,1.28]		0.81 [0.51,1.29]
Female	Ref.		Ref.
Age groups(in years)			
< 5	0.28[0.07,1.03]		0.31 [0.08,1.17]
5–15	0.89[0.37,2.11]		0.94 [0.39,2.27]
> 15	Ref.		Ref.
Duration of stay in the area			
> 3 years	Ref.		Ref.
1–3 years	2.54 [0.74,8.77]		2.33 [0.67,8.14]
7–12 months	5.59 [1.41,22.21]**		5.13 [1.26,20.92]**
< 6 months	7.66 [1.98,29.62]***		6.99 [1.68,29.00]***
ITN utilization			
Every night	Ref.		Ref.
Sometimes	1.64 [0.31,8.77]		1.77[0.32,9.73]
Never	1.70 [1.01,2.87]*		2.38[0.64,8.89]
Job			
Indoor	Ref.		Ref.
Outdoor	2.55 [1.20,5.42]**		2.93 [1.36,6.32]***
Migrant worker			
No	Ref.		Ref.
Yes	4.04 [2.10,7.77]***		14.68 [2.61,82.67]***
Irrigation status			
Non-irrigated		Ref.	Ref.
Irrigated		3.87 [1.81,8.25]***	4.09 [1.82,9.16]***
Season			
Dry		Ref.	Ref.
Wet		0.64[0.39,1.07]	0.77 [0.45,1.30]
Family size			
< 5		Ref.	Ref.
> 5		1.06 [0.57,1.95]	1.38 [0.72,2.63]
Number of ITN per household			
≥ Three		Ref.	Ref.
Two		0.44 [0.16,1.22]	0.40 [0.14,1.14]
One		1.30 [0.51,3.29]	1.02 [0.39,2.68]
Not available		1.64 [0.62,4.33]	0.62 [0.12,3.11]
Wall material			
Mud and wood		Ref.	Ref.
Corrugated iron		1.24 [0.56,2.75]	0.10 [0.02,0.52]***
Roof material			
Corrugated iron		Ref.	Ref.
Thatch		1.01 [0.45,2.24]	1.53 [0.64,3.70]
Number of sleeping rooms			
≥Three		Ref.	Ref.
Two		1.40 [0.43,4.55]	1.53 [0.45,5.13]
One		2.17 [0.66,7.09]	1.94 [0.56,6.71]

*p* <  = 0.01, **: *p* < 0.001***

### Measure of variability

In sugarcane growing (Arjo), the null model estimates of random effects variance revealed there was significant variation in microscopic infection between clusters [2.17, 95%CI 1.06–4.41, *p* < 0.001]. While, the individual level, cluster level and full model variance were higher than the null model and remained significant [2.25, 95%CI 1.10–4.60, *p* < 0.001], [2.43, 95%CI 1.11–5.33, *p* < 0.001] and [2.27, 95%CI 1.02–5.06, *p* < 0.001], respectively (Table 6). The variance partition coefficient (VPC) showed 39.71 variability in the odds of *Plasmodium* infection was due to cluster level risk factors after controlling individual level risk factor (model I). In the full model, 40.88 in the odds of *Plasmodium* infection variation was explained after adding individual and cluster level risk factors. MOR 4.07 was observed in the null model, individual-level predictors 4.18, cluster level predictors 4.43 and full 4.21 models were greater than one. This shows the presence of unexplained variation among the cluster. Akaike information criteria (AIC) identified the full model as the best fit.

**Table 6:** Measure of variation for *Plasmodium* infection by levels of risk factors using microscopy results, in Arjo and Gambella areas, Southwest Ethiopia: March 2019 and October 2019

Measure of variation	Sugarcane growing (Arjo)				Rice-growing (Gambella)			
	Null model	Model I	Model II	Full model	Null model	Model I	Model II	Full model
Variance [95%CI]	2.17 [1.06,4.41]	2.25 [1.10,4.60]	2.43 [1.11,5.33]	2.27 [1.02,5.06]	3.29 [1.75,6.19]	2.28 [1.22,4.23]	2.12 [1.14,3.93]	2.12 [1.12,4.02]
MOR	4.07	4.18	4.43	4.21	5.64	4.22	4.01	4.01
VPC/ICC%	39.71	40.61	42.53	40.88	49.98	40.89	39.16	39.23
AIC	856.01	851.49	798.21	793.50	964.97	897.63	926.39	887.59
VIF		1.05	1.49	1.60		1.47	2.19	2.79

*MOR* Median odds ratio, *VPC* Variance partition coefficient, *ICC* Interaclass correlation coefficience, *AIC* Akaike information criteria, *VIF* Variance inflation factor

In rice growing areas (Gambella), the cluster variance of the null model showed significant difference in *Plasmodium* infection [3.29, 95%CI 1.75–6.19, *p* = 0.001]. The individual, cluster level and full model variance was lower than the null model [2.28, 95%CI 1.22–4.23, *p* < 0.001, [2.12, 95%CI 1.14–3.93, p < 0.001 and [2.12, 95%CI 1.12–4.02, *p* < 0.001] respectively. The VPC showed 49.98% variability in the odds of *Plasmodium* infection was due to cluster level risk factors after controlling individual level risk factor (model I). In the full model, 39.23% in the odds of *Plasmodium* infection variation was explained after adding individual and cluster level risk factors. MOR 5.64 was observed in the null model, individual-level predictors 4.22, and 4.01 in cluster level and full models were greater than one. This shows the presence of unobserved variation among the cluster. Akaike information criteria (AIC) identified the full model as the best fit (Table 6).

## Discussion

In the present study, submicroscopic malaria transmission was influenced by irrigation and ITN utilization in sugarcane growing (Arjo) area. While in rice growing (Gambella) area, submicroscopic malaria was associated with irrigation, age, survey season, number of rooms in the house and length of stay in the area. However, microscopically detected malaria infection had shown seasonal pattern and affected by ITN utilization in sugarcane growing (Arjo). Irrigation, being a migrant worker, outdoor jobs, length of stay in the area, and house construction material were significant predictors of microscopically detected malaria. Interestingly *P. ovale* was detected in irrigated clusters of both sugarcane growing (Arjo) and rice growing (Gambella), southwest Ethiopia.

In this study, the prevalence of submicroscopic *Plasmodium* infection was 12.8% in rice growing areas (Gambella), a high transmission setting whereas, 1.2% in sugarcane growing areas (Arjo), low transmission setting. Several studies showed a range of submicroscopic prevalence in low transmission setting from 0 to 16.8 and 16.3 to 82.5 in high transmission setting respectively [42–44]. Studies in Ethiopia showed different prevalence of submicroscopic malaria 2.4 -19.2% [22, 45, 46]. Only *P. falciparum* and *P. vivax* caused submicroscopic malaria was reported in the aforementioned studies. It would be expected that in lower transmission areas the prevalence of both microscopic and sub-microscopic infections would be lower, as shown in your study (sugarcane growing, Arjo). In Arjo area, since 2018 primaquine (PQ) was extensively used targeting malaria elimination in low transmission setting. The use of PQ could lower the overall transmission, but it is not clear why that would only impact submicroscopic infections. Asymptomatic submicroscopic malaria is a major obstacle to malaria elimination due to its being a hidden reservoir and facilitate onward transmission. Therefore, malaria elimination requires targeting submicroscopic carriers [26].

*Plasmodium ovale* was reported in both study sites for the first time. All *P. ovale* infections were submicroscopic. Species identification and parasite distribution are important epidemiological parameter to understand a country’s parasite population that eventually informs the malaria control and elimination which interventions and diagnostic method to use. Previously *P. ovale* was reported in Ethiopia in 1969, but it was not reported until 2013 and 2015 from northwestern [47] and southeastern Ethiopia [48]. Two recent serological studies showed that there is evidence of the presence of *P. ovale* and *Plasmodium malariae* in different parts of the country [49, 50]. This indicate there is a need for a highly sensitive method to detect low-density parasite applicable in resource limited setting to achieve malaria elimination.

The finding of this study indicated submicroscopic malaria was disproportionately higher in school-age children in rice growing, Gambella area and the odds of submicroscopic *Plasmodium* infection among 5–15 years of children were 3.7 higher than individuals who were over the age of 15. This result was in line with research from other sources [16, 51–53]. Even though, high burden of malaria attributed to children under-five, they typically exhibit symptoms due to low malarial immunity. However, recent studies have reported a shift in malaria infection from children under the age of five to school-age children. The observed shift in prevalence might be due to decline of immunity as well as increased exposure to infective bites in school-age children.

This study also showed 4.9 and 3.1 fold increased submicroscopic infection by irrigation in both sugarcane growing areas (Arjo) and rice growing areas (Gambella), respectively. In Arjo, no significant increment of microscopically detected malaria was observed between sugarcane-irrigated and non-irrigated clusters however, rice irrigation showed fourfold increase in microscopically detected malaria infection than non-irrigated clusters. Moreover, microscopically detected malaria infection doubled in dry season (12.9%) than wet (6.7%) in rice irrigation clusters. Similar findings were also documented in two studies from east-central Tanzania [14, 16] and one study from Malawi [53]. On the other hand, high malaria prevalence was recorded in sugarcane irrigated than rice irrigated in northern Tanzania [17] and Ethiopia [15]. The discrepancy of the results might be due to the majority of study population were migrant workers in Tanzanian study and open gravity fed concrete was used in Ethiopian study. The possible explanetion for incerase of microscopically detected asymptomatic malaria in dry season in this study is 24.7% prevalence was observerd in migrant workers group.

Also, studies conducted in these study sites demonstrated that anopheline mosquito abundance was higher in rice-irrigated clusters than non-irrigated clusters and the sporozoite rate in rice-irrigated clusters was tenfold higher than non-irrigated clusters in rice-irrigated (Gambella) [3]. Whereas, in sugarcane growing (Arjo), the anopheline mosquito density was sevenfold higher in sugarcane-irrigated clusters than in non-irrigated clusters during wet season [32]. This could be the odor emanates from rice strongly attracts the female anopheline mosquitoes oviposition than the sugar cane [54, 55] and the microhabitats formed by the flooded type of rice irrigation is favourable for mosquito proliferation during the low malaria transmission season. However, in Arjo sugarcane plantation, large proportion of irrigation system was sprinkler water for seedlings. Thus, the result might suggest that rice irrigation had higher contribution for persistent malaria transmission in the area.

In the present study, microscopically detected malaria infection was 21-fold higher in the wet season compared to dry season. This finding was complemented by an entomological study that showed higher mosquito abundance in wet season than the dry season in Arjo [32]. In addition, a retrospective study which was conducted in Arjo showed higher malaria cases in wet season [56]. Variation in the prevalence of malaria in terms of season in irrigated sites was observed in other studies [13, 16, 29, 57]. The difference might be due to the type of irrigation and type of crop cultivated. However, 64% submicroscopic infection less likely to occur in dry survey season than wet survey season in rice growing, Gambella area.

Newly migrant population migrated in are more prone to malaria infection due to different reason. In rice growing (Gambella) study, being a migrant worker were 14.7 times more prone to microscopic malaria infection than the non-migrant residents. Half (50%) of this migrant population did not owned ITN during the study period while, only one fourth (22.9%) of the local residents did not owned ITN. In addition, individuals who stayed in the area for < 6 months and 7 to 12 months were seven and 5 times at risk of microscopic malaria infection than those who stayed for more than three years, respectively. Similarly, submicroscopic infection was higher in individual who stayed one to 3 years. This finding is in line with findings from several studies on migrant workers involved in commercial agricultural activities [7, 21, 58–60]. High proportion (24.7%) of seasonal migrant workers were microscopically detected asymptomatic malaria carriers during dry season in this study. This group repeatedly visit the study site for the job opportunity they have. This exposed them to infective bite, some of them might develop a partial immunity and they will carry the parasite for long duration and become persistent transmitter.

This study demonstrated a lower than expected protective effect of IRS. Reasons for this could be due to insecticide resistance, the insecticide dosage concentration, duration of insecticide on the wall, and other factors could affect the protective efficacy of IRS. Studies documented multiple insecticide resistance particularly to dichloro diphenyl trichloroethane (DDT) and pyrethroids at nationwide level and at the study site, Arjo and Gambella in Ethiopia [61–63]. However, no utilization of ITNs still significantly associated with *Plasmodium* infection in sugarcane growing (Arjo). The risk of submicroscopic and microscopic *Plasmodium* infection was 7.4-fold and fivefold increase in individuals who had no habit of utilization of ITN than households who used persistently, respectively. This result is in line with the results of different studies [16, 58, 64]. This can be explained by ITN provide as a physical barrier from the mosquito bite.

In this study, socio-economic factors, such as occupation and wall construction material, were also significantly associated with malaria. Study participants who had outdoor jobs were 2.9 times at higher risk of *Plasmodium* infection than those who had indoor jobs in rice growing (Gambella). This could be explained by malaria transmission occur at outdoor. However, wall construction material such as corrugated iron wall and number of sleeping rooms were 90% and 68% less likely to acquire microscopically detected and submicroscopic *Plasmodium* infection than their counterparts respectively in Gambella. Different studies showed poorly constructed house such as a wall or a roof, which had a gap or an open eaves may serve to a mosquito enter to a house than well-constructed houses [65–67].

## Limitation of the study

There are various limitation in this study that have not been included. These limitation includes lagged rainfall, enhanced vegetation index (EVI) and land surface temperature (LST) as a cluster level variable were not included in the data collection. Therefore, this calls for the need for further investigations in these areas.

## Conclusion

The study highlighted the potential importance of different irrigation practices impacting on submicroscopic malaria. Moreover, asymptomatic and submicroscopic malaria also adds a hidden burden to malaria control and elimination thus continues the ongoing transmission pattern. In this study, only *P. falciparum* and *P. vivax* were identified by microscopy however, *P. ovale* was detected by qPCR. This indicates there is a need of a highly sensitive diagnostic tool to detect the low-grade parasite in a resource-limited setting and to accelerate the progress of malaria elimination by detecting submicroscopic malaria reservoirs. Population movement such as migrant workers are also a challenge for malaria control and elimination efforts. Since their frequent mobility of this group, they have limited access to malaria diagnosis, treatment, and vector control measures. Therefore, it is crucial to specifically screen and treat this population upon arrival and departure in order to minimize residual malaria transmission. Length of stay in the area, and sociodemographic factor such as outdoor jobs and school-age children were shown risk factors for malaria infection. In addition, in sugarcane growing areas, poor ITN utilization and season were significant predictors. Therefore, strengthening malaria control and elimination efforts by ensuring adequate coverage and proper utilization of vector control tools, prompt diagnosis and treatment coupled with health education should be implemented in water resource development projects such as irrigation schemes.

### Supplementary Information

**Additional file 1: Table S1.** The clusters name and abbreviation of the sugarcane growing (Arjo) and rice growing (Gambella), Ethiopia: March 2019 and October2019. **Table S2.** Univariate mixed effect logistic regression analysis of individual level risk factors and malaria infection by microscopy in Arjo and Gambella, Ethiopia: March 2019 and October2019. **Table S3.** Univariate mixed effect logistic regression analysis of cluster level risk factors and malaria infection by microscopy in Arjo and Gambella, Ethiopia: March 2019 and October 2019. **Table S4.** Univariate analysis of GEE model submicroscopic malaria infection and risk factors in Arjo and Gambella, Ethiopia: March 2019 and October 2019

## Data Availability

The datasets used and/or analysed during the current study are available from the corresponding author on reasonable request.
