# Supplementary material for: Asymptomatic and submicroscopic malaria infections in sugar cane and rice development areas of Ethiopia
Source: Malar J. 2023 Nov 8;22:341. doi: 10.1186/s12936-023-04762-5 (PMC10634149; doi:10.1186/s12936-023-04762-5)
Supplement: Supplementary file 1 — Additional file 1: Table S1. The clusters name and abbreviation of the sugarcane growing (Arjo) and rice growing (Gambella), Ethiopia: March 2019 and October2019. Table S2. Univariate mixed effect logistic regression analysis of individual level risk factors and malaria infection by microscopy in Arjo and Gambella, Ethiopia: March 2019 and October2019. Table S3. Univariate mixed effect logistic regression analysis of cluster level risk factors and malaria infection by microscopy in Arjo and Gambella, Ethiopia: March 2019 and October 2019. Table S4. Univariate analysis of GEE model submicroscopic malaria infection and risk factors in Arjo and Gambella, Ethiopia: March 2019 and October 2019 [file 12936_2023_4762_MOESM1_ESM.pdf]

Table S1. The clusters name and abbreviation of the sugarcane growing (Arjo) and rice growing (Gambella), Ethiopia: March 2019 and October2019

Sugarcane growing (Arjo)		Rice growing (Gambella)	
**Irrigated**	**Non-irrigated**	**Irrigated**	**Non-irrigated**
Command 2 (CO2)	Ambelta (AMB)	Saudi Star Bravo (BRA)	Terkudi (TER),
Command 5 (CO5)	Bekelicha-Biftu (BEB)	Saudi Star GRC (GRC)	Village-12 (V12)
Command 8 (CO8)	Bildima-Daru (BLD)	Village-17 (V17)	Village-13 (V13)
Abote-Didessa1(AD1)	Chilalo-Bildima (CBL)		
Abote-Didessa2 (AD2)	Hunde-Gudina (HNG		
Chefe-Jalala (CHJ)	Soyema (HG1)		
Kerka (KER)	Sefra-Tabia (SFT)		
Beyima (BEM)			

Table S2. Univariate mixed effect logistic regression analysis of individual level risk factors and malaria infection by microscopy in Arjo and Gambella, Ethiopia: March 2019 and October2019

	**Sugarcane growing (Arjo)**		**Rice-growing (Gambella)**	
	**Model-I**		**Model-I**	
**Fixed effect**	OR[95%CI]	*p-value*	OR[95%CI]	*p-value*
**Sex**				
Male	1.03[0.65,0.1.61]	0.901	1.31[0.86,1.99]	0.209
Female	Ref.		Ref.	
**Age groups(in year):**				
< 5	1.14[0.63,2.07]	0.655	**0.24[0.10,0.58]**	**0.002**
5-15	0.89[0.50,1.61]	0.713	**0.30[0.16,0.55]**	**0.0001**
>15	Ref.		Ref.	
**Job**				
Indoor	Ref.		Ref.	
Outdoor	1.06[0.68,1.68]	0.783	**4.14[2.58,6.64]**	**0.0001**
**Migrant worker**				
No	Ref.		Ref.	
Yes	0.75[0.25,2.24]	0.604	**8.24[4.57,14.87]**	**0.0001**
**Duration of stay in the area**				
>3 years	Ref.		Ref.	
1-3 years	0.92[0.42,2.05]	0.848	1.14[0.41,3.17]	0.803
7-12 months	2.27[0.97,5.29]	0.058	1.53[0.52,4.50]	0.435
< 6 months	1.13[0.42,3.08]	0.807	**4.87[1.61,14.70]**	**0.005**
**ITN utilization**				
Every night	Ref.		Ref.	
Sometimes	**0.24[0.08,0.74]**	**0.013**	1.40[0.25,7.89]	0.701
Never	1.65[0.98,2.77]	0.059	**2.40[1.41,4.09]**	**0.001**

Table S3. Univariate mixed effect logistic regression analysis of cluster level risk factors and malaria infection by microscopy in Arjo and Gambella, Ethiopia: March 2019 and October2019

Fixed effect	Arjo (Sugarcane growing)		Gambella (Rice-growing)	
	**Model II**		**Model-II**	
	OR[95%CI]	*p- value*	OR[95%CI]	*p- value*
Cluster level				
**Irrigation status**				
Non-irrigated	Ref.		Ref.	
Irrigated	0.96[0.58,1.59]	0.869	**5.13[2.85,9.23]**	**0.0001**
**Season**				
Dry	Ref.		Ref.	
Wet	**11.56[4.77,28.00]**	**0.0001**	0.72[0.44,1.16]	0.178
**Family size**				
<5	Ref.		Ref.	
>5	1.49[0.88,2.51]	0.135	0.57[0.32,1.01]	0.056
**Household head level of education**				
≥Secondary	Ref.		Ref.	
Primary	0.84[0.34,2.11]	0.718	0.74[0.37,1.49]	0.402
No education	1.57[0.72,3.42]	0.260	0.87[0.47,1.64]	0.677
House construction material				
**Roof material**				
Corrugated iron	Ref.		Ref.	
Thatch	1.15[0.68,1.94]	0.596	**0.43[0.24,0.78]**	**0.005**
**Wall material**				
Mud and wood	Ref.		Ref.	
Corrugated iron	0.81[0.38,1.75]	0.601	**4.47[2.56,7.80]**	**0.0001**
**Number of sleeping rooms**				
≥ Three	Ref.		Ref.	
Two	0.86[0.42,1.78]	0.696	1.26[0.37,4.23]	0.707
One	0.81[0.38,1.73]	0.592	2.43[0.81,7.29]	0.112
Vector control measures				
**Number of LLIN per household**				
≥Three	Ref.		Ref.	
Two	0.69[0.34,1.40]	0.308	0.48[0.18,1.32]	0.158
One	0.52[0.24,1.14]	0.105	1.83[0.75,4.44]	0.181
Not available	1.04[0.52,2.07]	0.913	2.33[0.94,5.76]	0.066
**IRS sprayed the past 12 months**				
Yes	Ref.		Ref.	
No	0.96[0.58,1.58]	0.862	0.79[0.47,1.31]	0.356

Table S4. Univariate analysis of GEE model submicroscopic malaria infection and risk factors in Arjo and Gambella, Ethiopia: March 2019 and October 2019

	Arjo (Sugarcane growing)		Gambella (Rice-growing)	
**Characteristics**	OR[95%CI]	*p-value*	OR[95%CI]	*p-value*
**Sex**				
Male	1.44[0.57-3.67]	0.440	1.04[0.63-1.72]	0.868
Female	Ref.		Ref.	
**Age groups(in year):**				
<5	0.91[0.30-2.82]	0.877	1.04[0.41-2.64]	0.937
5-15	0.44[0.11-1.77]	0.250	**2.12[1.18-3.81]**	**0.012**
>15	Ref.		Ref.	
**ITN utilization**				
Every night	Ref.		Ref.	
Sometimes	**5.81[1.16-28.96]**	**0.032**	0.67[0.07-6.44]	0.732
Never	**8.06[2.26-28.69]**	**0.001**	1.05[0.59-1.85]	0.867
**Job**				
Indoor	Ref.		Ref.	
Outdoor	1.25[0.59-2.67]	0.558	0.83[0.50-1.40]	0.491
**Migrant worker**				
No	-	-	Ref.	
Yes	-	-	0.78[0.42-1.45]	0.440
**Duration of stay in the area**				
>3 years	-	-	Ref.	
1-3 years	-	-	**4.50[2.05-9.87]**	**0.0001**
7-12 months	-	-	3.21[0.75-13.71]	0.115
< 6 months	-	-	2.45[0.50-11.88]	0.266
**Irrigation status**				
Non-irrigated	Ref.		Ref.	
Irrigated	**5.12[1.47-17.75]**	**0.010**	**1.93[1.13-3.30]**	**0.015**
**Season**				
Dry	1.67[0.64-4.31]	0.291	**0.43[0.24-0.74]**	**0.002**
Wet	Ref.		Ref.	
**Family size**				
<5	Ref.		Ref.	
>5	1.13[0.44-2.87]	0.794	1.26[0.74-2.15]	0.399
**Household head level of education**				
≥Secondary	Ref.		Ref.	
Primary	1.25[0.32-4.79]	0.745	0.61[0.31-1.19]	0.147
No education	0.98[0.27-3.49]	0.973	0.69[0.37-1.27]	0.229
**Roof material**				
Corrugated iron	Ref.		Ref.	
Thatch	1.16[0.46-2.96]	0.750	0.72[0.41-1.27]	0.256
**Wall material**				
Mud and wood	Ref.		Ref.	
Corrugated iron	0.88[0.20-3.88]	0.866	1.09[0.64-1.87]	0.744
**Number of sleeping rooms**				
≥ Three	Ref.		Ref.	
Two	3.15[0.39-25.03]	0.278	0.69[0.28-1.71]	0.426
One	3.10[0.39-24.50]	0.282	**0.35[0.15-0.80]**	**0.013**
**Number of ITN per household**				
≥Three	-	-	Ref.	
Two	-	-	0.86[0.30-2.49]	0.781
One	-	-	0.93[0.35-2.52]	0.894
Not available	-	-	1.04[0.38-2.85]	0.942
**IRS sprayed the past 12 months**				
Yes	Ref.		Ref.	
No	1.59[0.62-4.08]	0.329	1.28[0.76-2.16]	0.352
